# Tumor-associated macrophages mediate resistance of EGFR-TKIs in non-small cell lung cancer: mechanisms and prospects

**DOI:** 10.3389/fimmu.2023.1209947

**Published:** 2023-08-15

**Authors:** Daoan Cheng, Kele Ge, Xue Yao, Banglu Wang, Rui Chen, Weiqing Zhao, Cheng Fang, Mei Ji

**Affiliations:** Departments of Oncology, the Third Affiliated Hospital of Soochow University, Changzhou, China

**Keywords:** NSCLC, EGFR-TKIs, resistance, tumor-associated macrophages, exosome

## Abstract

Epidermal growth factor receptor tyrosine kinase inhibitors (EGFR-TKIs) are the first-line standard treatment for advanced non-small cell lung cancer (NSCLC) with EGFR mutation. However, resistance to EGFR-TKIs is inevitable. Currently, most studies on the mechanism of EGFR-TKIs resistance mainly focus on the spontaneous resistance phenotype of NSCLC cells. Studies have shown that the tumor microenvironment (TME) also mediates EGFR-TKIs resistance in NSCLC. Tumor-associated macrophages (TAMs), one of the central immune cells in the TME of NSCLC, play an essential role in mediating EGFR-TKIs resistance. This study aims to comprehensively review the current mechanisms underlying TAM-mediated resistance to EGFR-TKIs and discuss the potential efficacy of combining EGFR-TKIs with targeted TAMs therapy. Combining EGFR-TKIs with TAMs targeting may improve the prognosis of NSCLC with EGFR mutation to some extent.

## 1 Introduction

### 1.1 Background

The epidermal growth factor receptor (EGFR) is one of the most frequently mutated driver oncogenes in non-small cell lung cancer (NSCLC), and EGFR mutation is found in approximately 50% of the Southeast Asian lung adenocarcinoma population (1). EGFR-tyrosine kinase inhibitors (EGFR-TKIs) such as first-generation EGFR-TKIs gefitinib or erlotinib have shown potent antitumor effects in advanced NSCLC patients with EGFR mutation (2). Osimertinib, a third-generation EGFR-TKI, has been approved as first-line therapy for advanced NSCLC patients with EGFR mutation due to its lower toxicity and stronger antitumor effects (3). However, resistance to EGFR-TKIs is inevitable, and disease progression occurs in most patients. The mechanisms of resistance to EGFR-TKIs are a current research focus in NSCLC. Several resistance mechanisms have been elucidated, including secondary mutations of EGFR, activation of bypass pathways, and histological transformation (4). The development of fourth-generation EGFR-TKIs targeting the EGFR C797S mutation is underway (5). In recent years, the resistance of EGFR-TKIs mediated by tumor-associated macrophages (TAMs) has received broad attention (**Table 1**). Previous studies have demonstrated that high infiltration of TAMs is significantly associated with an unfavorable prognosis in NSCLC patients treated with EGFR-TKIs (6–9).

**Table 1:** Mechanisms of TAMs mediated resistance to EGFR-TKIs.

Mechanisms	References
Activating bypass pathways	
AKT/mTOR pathway	34
AKT, ERK1/2 and STAT3 pathways	17, 59
LncRNA-MSTRG.292666.16/miR-6836-5p/MAPK8IP3 pathway	35
NF-κB/RELB pathway	36
Suppressing T cells	
NOS and PD-L1 pathways	91
M2-like polarization	
Lipid metabolism pathways	103
STAT3/IL-4 pathway	107
LncRNA SOX2-OT/miR-627-3p/Smads pathway	114
Modulating tumor cell phenotypes	
Stabilizing tumor cell phenotype	115
Promoting the EMT	129, 130

TAM, tumor-associated macrophage; mTOR, mammalian target of rapamycin; RELB, v-rel reticuloendotheliosis viral oncogene homolog B; NOS: nitric oxide synthase; PD-L1, programmed cell death 1 ligand 1; LncRNA SOX2-OT, long non-coding RNA SOX2 overlapping transcript; EMT, epithelial-mesenchymal transition.

### 1.2 TAMs in NSCLC

The origin of TAMs in NSCLC is multifaceted, involving both tissue-resident macrophages (TRMs) and monocyte-derived macrophages (MDMs) (10). And TRMs can be classified into lung alveolar macrophages (LAMs) and interstitial macrophages (IMs) based on their anatomical locations. TRMs are present during embryonic development and can self-renew locally, independent of the hematopoietic system (11). They are crucial in coordinating tissue remodeling and maintaining tissue integrity (11). MDMs originate from the hematopoietic system, and many can be observed in inflammatory lesions (12). TAMs from different sources can promote the progression of NSCLC (13). TRMs mainly contribute to tumor generation, while MDMs primarily participate in tumor metastasis (13).

Macrophages can generally be classified into M1 and M2 types based on their polarization status (14, 15). M1-like macrophages secrete pro-inflammatory factors, such as tumor necrosis factor-α (TNF-α), interleukin-1β (IL-1β), IL-6, IL-12, and IL-23, to participate in antigen presentation and play a role in immune surveillance (16). And M2-like macrophages secrete inhibitory factors, such as IL-10 and transforming growth factor-β (TGF-β), and have weak antigen-presenting ability (16). TAMs mainly exhibit the M2-like macrophage phenotype (17) and are closely associated with resistance to anti-tumor drugs in various solid tumors, including NSCLC (6, 18–20). Additionally, TAMs exhibit both inter- and intra-tumor heterogeneity. High infiltration of TAMs has been linked to unfavorable prognosis in pancreatic cancer (21), bladder cancer (22), and malignant glioma (23). But in some instances, such as ovarian (24) and colorectal cancers (25), it is associated with a more favorable outcome. In the NSCLC investigation, a high TAMs infiltration level within tumor islets was associated with a favorable prognosis. In contrast, a high level of TAMs infiltration within tumor stroma was linked to unfavorable prognosis (26, 27). The heterogeneity of TAMs in NSCLC may be attributed to tumor hypoxia and the spatial distribution of TAMs within the tumor microenvironment (TME) (28).

### 1.3 Effects of EGFR-TKIs on TAMs

Jia et al. (29) investigated the impact of EGFR-TKIs on the TME in NSCLC from a dynamic perspective. During early-stage treatment, EGFR-TKIs can increase the infiltration of CD8^+^T cells and dendritic cells (DC) in TME while inhibiting the infiltration of Foxp3^+^ regulatory T cells (Tregs) and M2-like polarization of TAMs (29). However, with the continuation of treatment, the immune-activated TME gradually dissipated while the proportion of immunosuppressive cells, myeloid-derived suppressor cells (MDSCs), progressively increased (29). Notably, there was a significant increase in CD86^+^ macrophage expression driven by EGFR during the initial phase of EGFR-TKIs treatment, which exhibited robust antigen presentation capabilities (29). However, the gradual accumulation of M2-like TAMs, MDSCs, and Tregs during treatment hindered the antitumor immune effects of DC and T cells (29–31).

### 1.4 Aims

**Figure 1:**
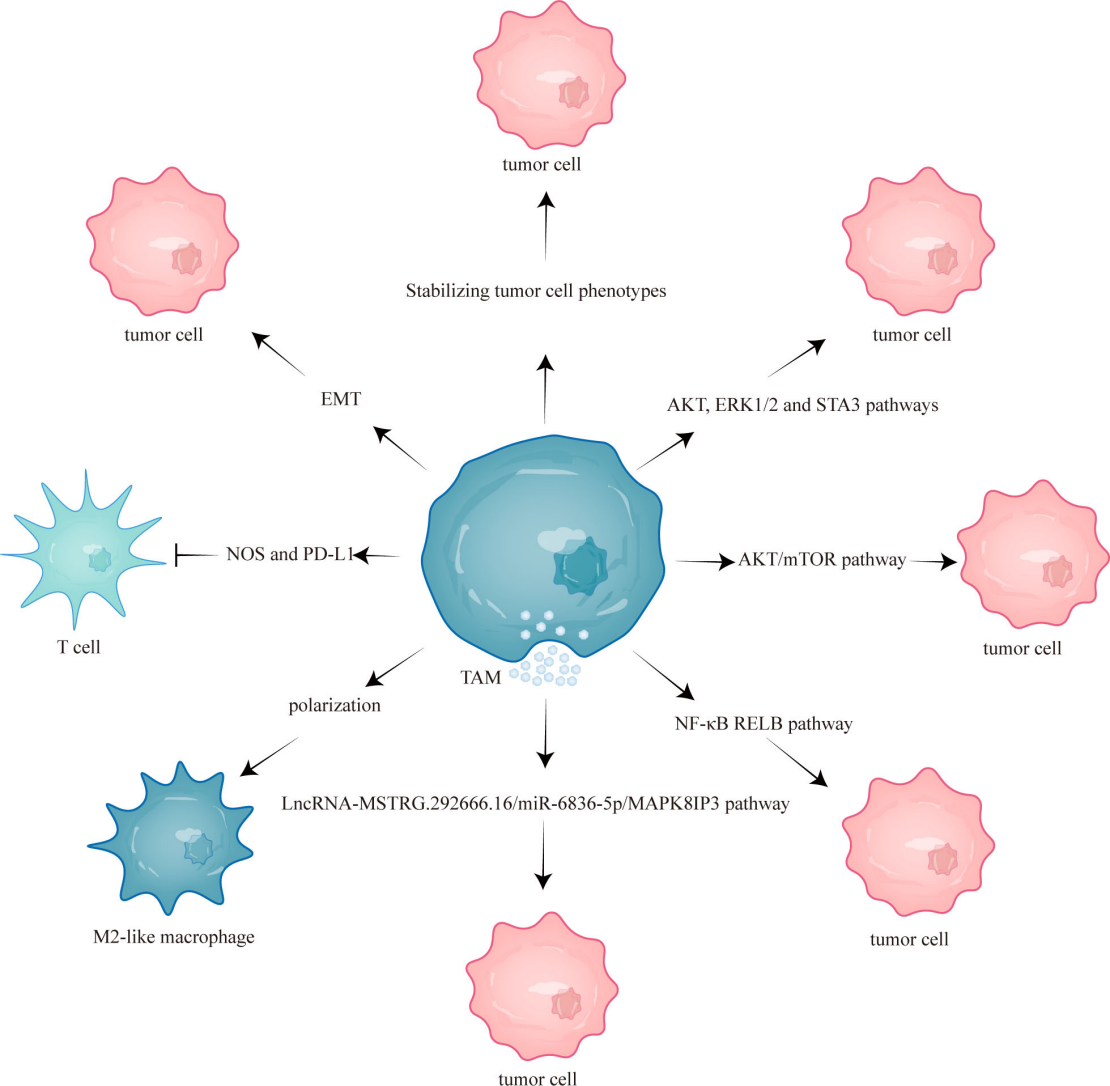
TAMs mediated EGFR-TKIs resistance through different mechanisms. TAM, tumor-associated macrophage; mTOR, mammalian target of rapamycin; NOS, nitric oxide synthase; EMT, epithelial-mesenchymal transition; RELB, v-rel reticuloendotheliosis viral oncogene homolog B; PD-L1, programmed cell death one ligand 1.

This study aims to comprehensively review the current mechanisms underlying TAM-mediated resistance to EGFR-TKIs and discuss the potential efficacy of combining EGFR-TKIs with targeted TAMs therapy (**Figure 1**). Combining EGFR-TKIs with TAMs targeting may improve the prognosis of NSCLC with EGFR mutation to some extent.

## 2 TAMs mediate EGFR-TKIs resistance by activating bypass pathways

### 2.1 Background

Activation of phosphoinositide 3-kinase (PI3K)/protein kinase B (AKT) and mitogen-activated protein kinase (MAPK) signaling pathways compensates for the inhibition of EGFR signaling by EGFR-TKIs, promoting resistance of EGFR-TKIs (32). Yuan et al. (33) showed that TAMs can affect the biological behavior of lung adenocarcinoma cells by activating the PI3K/AKT pathway. This suggests that TAM-mediated EGFR-TKIs resistance may be closely related to the activation of bypass pathways. Furthermore, several studies have demonstrated that TAMs contribute to the resistance of EGFR-TKIs by activating bypass pathways, such as AKT/mammalian target of rapamycin (mTOR) pathway (34), AKT pathway (17), extracellular signal-related kinases 1 and 2 (ERK1/2) pathway (17), signal transducer and activator of transcription 3 (STAT3) pathway (17), LncRNA-MSTRG.292666.16/miR-6836-5p/MAPK8IP3 pathway (35) and atypical nuclear factor-κB (NF-κB)/v-rel reticuloendotheliosis viral oncogene homolog B (RELB) pathway (36).

### 2.2 AKT/mTOR pathway

EGFR-TKIs can increase the content of serum chemokine (C-C motif) ligand 2 (CCL2) (29), which plays an essential role in the process of EGFR-TKI resistance (8). CCL2 in the TME can recruit macrophages (37–39). Xiao et al. (34) showed that gefitinib resistance cell lines increased the release of CCL2 by decreasing the expression of β-catenin protein. Furthermore, tumor cells recruit more M2-like macrophages by releasing CCL2, and these macrophages promote gefitinib resistance by activating the AKT/mTOR pathway (34). As a serine/threonine kinase, mTOR has a catalytic domain similar to PI3K and is considered an atypical protein kinase in the PI3K-related kinase family (40). Through various mechanisms, including activation of growth factor receptor pathway, inhibition of autophagy, and influence on lipid metabolism pathway et al., mTOR could promote tumor development, metastasis, and drug resistance (41, 42). The rapamycin analogs, which inhibit mTOR, have been approved for treating renal cell carcinoma, while several other mTOR inhibitors are currently in development (40).

#### 2.2.1 Prospects

Preclinical studies (43–50) have shown that mTOR inhibitors can improve the resistance of NSCLC to EGFR-TKIs. For example, Wang et al. (51) showed that the combination of ferumoxytol and CpG oligodeoxynucleotide 2395 could effectively suppress EGFR and its downstream AKT/mTOR signaling pathway, thereby enhancing the antitumor activity of macrophages in NSCLC with EGFR mutation. Qu et al. (52) employed a combination of MEK1/2 inhibitor AZD6244 and PI3K/mTOR inhibitor BEZ235 to improve gefitinib resistance in a xenograft model of NSCLC. However, Moran et al. (53) showed that afatinib, in combination with mTOR inhibitor sirolimus, did not show the expected anti-tumor effect. The toxicity was not tolerable in NSCLC patients with EGFR-TKIs resistance. The intricate resistance mechanism of EGFR-TKIs may account for the limited antitumor efficacy. This implies the necessity of identifying NSCLC patients who are responsive to mTOR inhibitors. Notably, altering the administration mode of mTOR inhibitors to target the TME in NSCLC might alleviate the adverse effects of combination therapy. In conclusion, further exploration is warranted for the combination of mTOR inhibitors and EGFR-TKIs in EGFR-mutated NSCLC based on available evidence (54, 55).

### 2.3 AKT, ERK1/2 and STAT3 pathways

Exosomes are extracellular vesicles ranging in size from 30 to 150nm, capable of transporting nucleic acids or proteins derived from maternal cells and facilitating intercellular communication (56). Exosomes play a crucial role in the pathogenesis, progression, and metastasis of tumors (57). Yuan et al. (17) investigated the contribution of TAM-derived exosomes to EGFR-TKI resistance and demonstrated that these exosomes could impede the antitumor efficacy of gefitinib. Further protein expression analysis confirmed that TAMs-derived exosomes mediated EGFR-TKIs resistance by activating AKT, ERK1/2, and STAT3 signaling pathways (17). On the other hand, previous studies have shown that epiregulin (EREG), as a ligand for EGFR, can promote the progression of NSCLC (58). EREG-enriched macrophages induce gefitinib and erlotinib resistance by inducing AKT phosphorylation in a human epidermal growth factor receptor 2 (HER-2)-dependent manner (59).

#### 2.3.1 Prospects

The abnormal activation of the AKT pathway is closely related to the resistance of EGFR-TKIs in NSCLC (60, 61). Several studies (62–67) have shown that inhibition of the AKT pathway can improve the resistance of EGFR-TKIs in NSCLC. For example, Lai et al. (68) demonstrated that Polyphyllin I can reverse osimertinib resistance by regulating the PI3K/AKT pathway in NSCLC. Wang et al. (69) showed that combination therapy with gefitinib and miR-30a-5p could overcome acquired resistance to EGFR-TKIs by regulating the PI3K/AKT pathway in NSCLC. However, Clément-Duchêne et al. (70) showed no improvement in progression-free survival (PFS) and overall survival (OS) for EGFR-mutated NSCLC treated with enzastaurin (an oral AKT inhibitor) combined with erlotinib compared to erlotinib alone in a phase II study. This finding contradicts previous preclinical studies and warrants further investigation to identify the subset of NSCLC patients who may benefit from AKT inhibitors. Additionally, TAM-induced AKT phosphorylation is closely associated with HER-2 (59), suggesting that the use of HER-2 inhibitors may improve resistance to EGFR-TKIs in NSCLC (71, 72). Consistent with this hypothesis, Peng et al. (73) have developed a trastuzumab-modified, mannosylated liposome system that effectively targets M2-type TAMs and HER-2 positive NSCLC cells to overcome EGFR-TKIs resistance mediated by the EGFR T790M mutation. Importantly, HER-2 and HER-3 belong to the HER family and have highly similar structures and biological functions (74). Vicencio et al. (75) demonstrated that osimertinib combined with HER-3 antibody therapy could enhance the antitumor effect in NSCLC. Therefore, in addition to directly inhibiting the AKT pathway, combining HER2 or HER3 inhibitors may be a therapeutic strategy for improving the efficacy of EGFR-TKIs.

### 2.4 LncRNA-MSTRG.292666.16/miR-6836-5p/MAPK8IP3 pathway

Non-coding RNAs (ncRNAs), including circular RNA (circRNA), long ncRNA (lncRNA), and microRNA (miRNA) et al., play an essential role in the initiation and progression of cancer (76). Deng et al. (77) analyzed the serum exosomal-lncRNAs of osimertinib resistant patients and found that the knock of lncRNA MSTRG.292666.16 can improve the osimertinib resistance in NSCLC cells. Furthermore, Wan et al. (35) showed that TAM-derived exosomes promote osimertinib resistance by activating MSTRG.292666.16/miR-6836-5p/1MAPK8IP3 signaling pathway in NSCLC.

#### 2.4.1 Prospects

TAM-derived exosomes play a crucial role in mediating resistance to EGFR-TKIs. Unfortunately, there is a lack of effective methods to target these exosomes. Further investigation is warranted to refrain from the biogenesis of TAM-derived exosomes or impede the binding of exosomes to tumor cells.

### 2.5 NF-κB/RELB pathway

In pathological conditions like cancer, myeloid cells may transform myeloid-derived suppressor cells (MDSCs), contributing to tumor metastasis and conferring resistance to anti-cancer drugs (78). MDSCs play a crucial role in promoting immunosuppression and inducing the generation of regulatory T cells within the TME (79). Feng et al. (36) suggested that S100A9^+^ MDSC (a subset of monocytic MDSC) derived macrophages induce gefitinib resistance *via* NF-κB/RELB pathway.

#### 2.5.1 Prospects

The oncogenic role of NF-κB has been reported (80). Notably, NF-κB can facilitate the epithelial-mesenchymal transition (EMT) of tumor cells, which may constitute one of the potential mechanisms by which TAMs mediate resistance to EGFR-TKIs (80). Targeting NF-κB has been reported to improve EGFR-TKIs resistance potentially. For example, Yeo et al. (81) improved acquired resistance to EGFR-TKIs by inhibiting NF-κB and activation-induced cytidine deaminase (AICDA) in NSCLC. Liu et al. (82) reported that Liver X receptor ligands could induce apoptosis in EGFR-TKIs resistant cells by inhibiting the AKT-NF-κB pathway in NSCLC. On the other hand, RELB can upregulate the expression of programmed cell death one ligand 1 (PD-L1) and facilitate immune evasion in prostate cancer (83). Previous studies (84) have shown that PD-L1 expression is also increased in NSCLC patients after developing resistance to EGFR-TKIs, and RELB may up-regulate PD-L1 expression following EGFR-TKIs resistance in NSCLC. Up-regulation of PD-L1 promotes an immunosuppressive TME, which may also be one potential mechanism for EGFR-TKI resistance (84). Therefore, RELB may be a potential target for improving EGFR-TKIs resistance in NSCLC.

## 3 TAMs mediate EGFR-TKIs resistance by suppressing T cells

### 3.1 Background

The EGFR signal can reduce chemokine (C-X-C motif) ligand 10 (CXCL10) and CCL5 by reducing interferon regulatory factor-1 (85). EGFR-TKIs can induce an interferon response in NSCLC, and the efficacy of EGFR-TKIs is influenced by immune activation (86). Previous studies (87–89) have shown that macrophages can promote chemotherapy resistance by inhibiting T-cell-mediated responses. Similarly, TAMs mediate T cell inhibition in the TME (90), which causes resistance to EGFR-TKIs related to TAMs (91).

### 3.2 TAMs inhibit T cells by expressing inducible nitric oxide synthase and PD-L1

Stimulator of interferon genes (STING) regulates the human immune system (92). Lin et al. (91) demonstrated that the enrichment of TAMs impedes T cell activation in NSCLC patients treated with osimertinib. The immunosuppressive TME attenuates the efficacy of EGFR-TKIs in anti-tumor therapy (91). Reprogramming macrophages with STING agonist, MSA-2, can restore T cell activation and reverse osimertinib resistance (91). This implies that the combination of EGFR-TKIs and STING agonists can potentiate the antitumor effects of EGFR-TKIs. In addition, Lin et al. (91) demonstrated that TAMs may mediate T-cell inhibition by up-regulating the expression of inducible nitric oxide (NO) synthase and PD-L1. Studies have shown that NO can promote cisplatin resistance in NSCLC and inhibit T cell proliferation (93, 94). Upregulation of PD-L1 expression in TAMs can increase immunosuppression and tumor aggressiveness in NSCLC (95, 96). These mechanisms provide targets for reactivating T cells in TME. However, Lin et al. (91) did not rule out the possibility that other cells may also be involved in anti-tumor immunity when stimulated by STING agonists, such as dendritic cells and endothelial cells (97, 98).

### 3.3 Prospects

Further deliberation is warranted on strategies to enhance T cell infiltration in the TME of NSCLC. Immune checkpoint inhibitors (ICIs) can potentially induce M1 polarization of TAMs and reactivate T cells within the TME (99). Theoretically, the combination therapy of ICIs and EGFR-TKIs may enhance the efficacy of EGFR-TKIs in NSCLC. However, the combination of ICIs and EGFR-TKIs has been found to result in intolerable toxicity during clinical trials (100). Strategies to enhance T cell infiltration in the TME of NSCLC, such as reprogramming TAMs or reducing their infiltration, should be developed for clinical implementation.

## 4 TAMs mediate EGFR-TKIs resistance through M2-like polarization of macrophage

### 4.1 Lipid metabolism pathways

**Figure 2:**
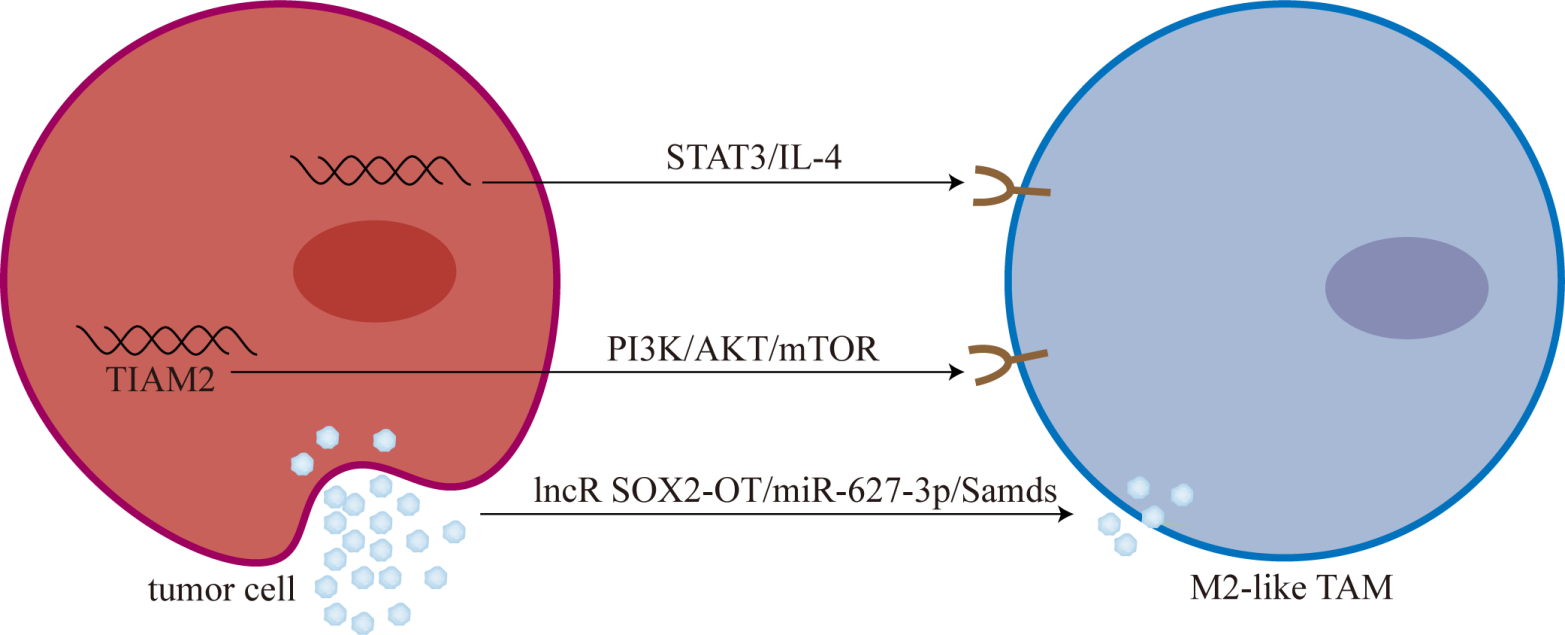
Tumor cells promoted M2-like polarization of TAMs. M2-like TAM: M2-like tumor-associated macrophage; mTOR, mammalian target of rapamycin; lncR SOX2-OT, long non-coding RNA SOX2 overlapping transcript; Smads, drosophila mothers against decapentaplegic proteins.

Lipid metabolism is closely related to TAMs polarization (101). Chen et al. (102) showed that overexpression of sterol regulatory element-binding protein 1 (SREBP1) can mediate osimertinib resistance. Furthermore, Liang et al. (103) analyzed the role of 9 genes related to lipid metabolism in osimertinib resistance. They found that T-cell lymphoma invasion and metastasis 2 (TIAM2) can induce TAMs M2-like polarization mediated osimertinib resistance through PI3K/AKT/mTOR signaling pathway (**Figure 2**).

#### 4.1.1 Prospects

Targeting lipid metabolic pathways to cause repolarization of TAMs is a feasible approach to improve resistance to EGFR-TKIs. Jin et al. (104) found that simvastatin can mediate TAMs repolarization by targeting cholesterol metabolism. Yin et al. (105) developed a dual-targeting liposomal system for the codelivery of simvastatin/gefitinib to treat NSCLC with brain metastases. Dual-targeting liposomal system with modification of anti-PD-L1 nanobody and transferrin receptor-binding peptide T12 can enter the blood-brain barrier to reverse EGFR T790M mutation-mediated resistance *via* TAMs repolarization (105).

### 4.2 STAT3/IL-4 pathway

Chen et al. (106) found that T790M-cis-L792F mutation is one of the mechanisms of osimertinib resistance. And Sun et al. (107) found that the expression and secretion of IL-4 increased in T790M-cis-L792F mutant cells, promoting the M2-like polarization of TAMs. Furthermore, Sun et al. (107) demonstrated that TAMs M2-like polarization is one of the downstream mediators of the STAT3/IL-4 signaling pathway, and blocking STAT3 with SH-4-54 and IL-4 with dupilumab can reverse osimertinib resistance to some extent.

#### 4.2.1 Prospects

Targeting STAT3 could be a promising strategy for overcoming resistance to EGFR-TKIs (108). Park et al. (109) showed that the root extract of Scutellaria baicalensis can induce apoptosis in EGFR-TKIs resistant NSCLC by inhibiting STAT3. Shu et al. (110) reversed afatinib resistance in NSCLC by knocking down lncRNA BLACAT1 by regulating STAT3 signaling. In addition, it has been previously reported that aberrant activation of STAT3 can promote M2-like polarization of macrophages (111). Lu et al. (111) showed that gefitinib combined with STAT3 inhibitor and anti-CD47 monoclonal antibody could reprogram TAMs and ameliorate acquired resistance to gefitinib in NSCLC. Small molecule inhibitors targeting STAT3 have shown preliminary antitumor effects (112, 113). Further investigation into STAT3 and IL-4 as potential targets is warranted to overcome resistance to EGFR-TKIs.

### 4.3 LncRNA SOX2-OT/miR-627-3p/Smads pathway

Recently, Zhou et al. (114) found that long non-coding RNA SOX2 overlapping transcript (lncRNA SOX2-OT) is highly expressed in exosomes derived from NSCLC cells. Subsequently, exosomal lncRNA SOX2-OT can promote M2-like polarization of TAMs and promote EGFR-TKIs resistance (114). Mechanistically, lncRNA SOX2-OT promotes M2-like polarization of TAMs by increasing the expression of drosophila mothers against decapentaplegic proteins (Smads) through sponging miR-627-3p (114).

#### 4.3.1 Prospects

lncRNA SOX2-OT/miR-627-3p/Smads axis represents a promising target for reprogramming TAMs. However, there still needs to be more feasible approaches to target this pathway.

## 5 TAMs mediate EGFR-TKIs resistance by modulating tumor cell phenotypes

### 5.1 Stabilizing tumor cell phenotypes

Zhao et al. (115) treated NSCLC cells with gefitinib and subsequently co-cultured them with macrophages to mimic the behavior of migrating macrophages. Migrating macrophages contributed to gefitinib resistance by stabilizing tumor cell phenotypes before macrophage polarization. Additionally, Zhao and colleagues (115) postulated that the upregulation of vimentin mediated by TGF-β might also account for the accelerated acquisition of gefitinib resistance in NSCLC cells.

#### 5.1.1 Prospects

Reducing the recruitment of TAMs or depleting the TAMs in the TME of NSCLC may be a potential approach to improve EGFR-TKIs resistance. CCL2-chemokine (C-C motif) receptor 2 (CCR2) signaling and the colony-stimulating factor 1(CSF1)-CSF1 receptor (CSF1-CSF1R) axis are potential therapeutic targets (116, 117). For example, previous studies (118) have shown that CSF1R inhibitors can deplete M2 macrophages in the TME. Sidorov et al. (119) demonstrated that the combination therapy of erlotinib and MLN0128 (an mTOR inhibitor) effectively reduces the infiltration of immunosuppressive chemokines, such as CCL2 and periostin, as well as TAMs in the TME of glioblastoma, leading to a significant improvement in survival outcomes for glioblastoma mice. Schmall et al. (120) demonstrated that inhibiting the recruitment of TAMs and promoting their M1-like polarization through CCR2 inhibition can effectively inhibit lung cancer progression. In addition, targeting surface receptors such as CD52, scavenger receptor-A, folic acid receptor-β, and CD206 represents potential approaches for depleting TAMs (121). Future research endeavors should investigate the clinical applications of these protocols in NSCLC.

### 5.2 Promoting the EMT

EMT, the process of epithelial-to-mesenchymal transition, plays a crucial role in physiological processes such as wound healing, development, and stem cell behavior (122). However, it is closely associated with tumorigenesis, tumor progression, and drug resistance under pathological conditions (123). Importantly, EMT is one of the mechanisms of acquired resistance to EGFR-TKIs (124). Approaches to overcome EGFR-TKI resistance in NSCLC by reversing EMT are currently under investigation, including the targeting of CD70, cyclin-dependent kinase 7 (CDK7), lipid metabolism pathways, and fibroblast growth factor receptor 1 (FGFR1) (125–128). Several studies (129, 130) have revealed that TAMs can promote the EMT of tumor cells. Bonde et al. (129) showed that TAMs promote tumor EMT through TGF-β signaling and activation of the β-catenin pathway in NSCLC. And Shen et al. (130) demonstrated that inhibition of TAMs can reverse tumor EMT in NSCLC.

#### 5.2.1 Prospects

Further investigation is warranted to target TAMs to reverse EMT in EGFR-TKI-resistant cells. Reprogramming TAMs to reduce the secretion of pro-EMT signals, such as TGF-β, may represent a promising strategy. Consistent with this hypothesis, Jin et al. (104) showed that targeting lipid metabolism could improve EMT-related drug resistance by reprogramming TAMs in NSCLC.

## 6 Discussion

Resistance to EGFR-TKIs remains a global challenge, and exploring new methods to enhance the efficacy of EGFR-TKIs is imperative in NSCLC. This review summarizes the multiple mechanisms of TAM-mediated EGFR-TKIs resistance in NSCLC, including activation of bypass pathways, inhibition of T cell activity, M2-like polarization, and regulation of tumor cell phenotypes. Several pertinent issues warrant discussion.

Inhibiting the TAMs-related bypass pathway may be a potential approach to improving resistance to EGFR-TKIs in NSCLC (**Table 2**). The significance of the mTOR-related pathway in enhancing resistance to EGFR-TKIs warrants reiteration. Previous studies (131) have shown that high expression of mTOR correlates with a diminished therapeutic response to erlotinib in NSCLC. TAMs can induce resistance to EGFR-TKIs by activating the AKT/mTOR signaling pathway directly (34). Additionally, mTOR-related pathways may mediate EMT-related EGFR-TKIs resistance (132). Zhang et al. (133) demonstrated that MTI-31, an inhibitor of mTORC1/2, effectively impedes the progression and EMT of NSCLC while simultaneously enhancing antitumor immunity. Significantly, the PI3K/AKT/mTOR signaling pathway can also facilitate M2-like polarization of TAMs to promote EGFR-TKIs resistance (103). Based on this existing evidence, combination therapy involving mTOR inhibitors and EGFR-TKIs may improve resistance to EGFR-TKIs by blocking multiple resistance signals. However, current clinical trials have demonstrated that the combination therapy does not yield a superior clinical response compared to EGFR-TKIs monotherapy, and its toxicity profile is challenging to manage (53). Further experimentation is warranted to elucidate this phenomenon in the future. On the other hand, targeting STAT3 may represent a promising strategy to enhance the efficacy of EGFR-TKIs by overcoming TAM-mediated resistance. TAMs-derived exosomes can mediate EGFR-TKIs resistance by activating STAT3 signaling pathway (17). Moreover, STAT3 also plays a crucial role in promoting the M2-like polarization of TAMs (111). Combining STAT3 inhibitors with EGFR-TKIs inhibits drug resistance mediated by exosomes derived from TAMs and reprograms TAMs. Previous studies (134–136) have shown the potential of STAT3 inhibitors in combination with EGFR-TKIs for anti-tumor therapy. W2014-S, a novel STAT3 inhibitor, can significantly enhance the anti-tumor effect of EGFR-TKIs in TKI-resistant NSCLC (137). Wang et al. (138) demonstrated that the STAT3 inhibitor BBI608 could potentiate the anti-tumor efficacy of EGFR-TKIs by modulating the ROR1/ABCB1/P53 signaling pathway.

**Table 2:** Strategies for improving resistance to EGFR-TKIs by targeting TAMs.

Strategies	Up/Down	Targets	References
Dictamnine	Down	PI3K/AKT/mTOR and MAPK pathways	43
Torin2	Down	AKT/mTOR pathway	44
Temsirolimus	Down	mTOR	45
BEZ235	Down	PI3K/mTOR pathway	46, 49, 52
Active fraction (HS7) from Taiwanofungus camphoratus	Down	AKT-mTOR, ERK and STAT3 pathways	47
Everolimus	Down	mTOR	45,48, 55
Ku-0063794	Down	mTOR	50
Ferumoxytol and CpG oligodeoxynucleotide 2395	Down	EGFR and AKT/mTOR pathways	51
T0901317 and GW3965	Down	AKT	62
Bufalin	Down	MET/PI3K/AKT Pathway	63
Chloroquine	Down	AKT	64
Norcantharidin	Down	MET/PI3K/AKT Pathway	65
MiR-30a-5p	Down	PI3K/AKT Pathway	66, 69
BMS-708163	Down	PI3K/AKT Pathway	67
Polyphyllin I	Down	PI3K/AKT Pathway	68
anti-HER-3 antibody	Down	HER-3 and PI3K/AKT Pathway	71
anti-HER-3 antibody	Up	STING	75
HECrossMAb	Down	PI3K/AKT Pathway	72
Gefitinib and Vorinostat	Up	M1-like polarization of TAMs	73
Cosuppression of NF-κB and AICDA	Down	NF-κB and AICDA	81
Liver X receptors agonist	Down	AKT and NF-κB	82
STING agonist MSA-2	Up	M1-like polarization of TAMs	91
Simvastatin	Up	M1-like polarization of TAMs	104, 105
The Root Extract of Scutellaria baicalensis	Down	STAT3	109
Knockdown of lncRNA BLACAT1	Down	STAT3	110
STAT3 inhibitor and an anti-CD47 monoclonal antibody	Up	M1-like polarization of TAMs	111
Simvastatin	Down	EMT	104
MTI-31	Down	EMT	133
W2014-S	Down	STAT3	137
BBI608	Down	STAT3	138

EGFR-TKIs, epidermal growth factor receptor tyrosine kinase inhibitors; TAMs, tumor-associated macrophages; PI3K, phosphoinositide 3-kinase; AKT, protein kinase B; mTOR, mammalian target of rapamycin; MAPK, mitogen-activated protein kinase; ERK, extracellular signal-related kinases; STAT3, signal transducer and activator of transcription 3; MET, mesenchymal to epithelial transition factor; HER-3, human epidermal growth factor receptor 3; STING: Stimulator of interferon genes; NF-κB, nuclear factor-κB; AICDA, activation-induced cytidine deaminase; EMT, epithelial-mesenchymal transition.

Reprogramming TAMs is a crucial strategy for improving the resistance to EGFR-TKIs in NSCLC. It is worth mentioning that reprogramming TAMs can enhance the efficacy of EGFR-TKIs through a variety of mechanisms, including inhibition of TAM-related drug resistance pathway (34), reactivation of T cells in the TME (91), and reversal of EMT of tumor cells (104). STING (91), lipid metabolic pathways (101), mTOR (34), Smads (114), IL-4 (107), and STAT3 (111) have been reported as targets for reprogramming TAMs in NSCLC. In addition, other strategies for reprogramming TAMs are currently under investigation, which may offer insights into improving resistance to EGFR-TKIs in NSCLC. Parayath et al. (139) reprogrammed TAMs by intraperitoneal injection of Hyaluronic Acid-Based Nanoparticles Encapsulating MicroRNA-125b in NSCLC. Sarode et al. (140) reprogrammed TAMs by targeting the β-catenin/FOSL2/ARID5A signaling pathway in lung cancer. Future research should investigate innovative approaches to reprogramming TAMs in NSCLC with EGFR mutation.

Finally, reducing the number of TAMs in the TME of EGFR-mutant NSCLC, either by inhibiting TAM recruitment or depleting TAMs, may represent a promising strategy to overcome resistance to EGFR-TKIs. The clinical applicability of these methods warrants further investigation (116, 117).

## 7 Conclusions

TAMs mediate EGFR-TKIs resistance in NSCLC through various mechanisms, including activation of bypass pathways, inhibition of T cell activity, M2-like polarization, and regulation of tumor cell phenotypes. In the future, developing therapeutic regimens that target TAMs, such as interfering with TAM-related pathways, reducing infiltration of TAMs, and reprogramming the macrophage phenotype, could enhance the anti-tumor effect of EGFR-TKIs.

## Author contributions

DC, KG, and XY wrote the original draft of the article and drew the illustration. RC, BW, WZ, CF, and MJ contributed to the conceptualization and revised the draft. All authors participated in the revision of the manuscript. All authors contributed to the article and approved the submitted version.

## References

[1] Recondo G Facchinetti F Olaussen KA Besse B Friboulet L. Making the first move in egfr-driven or alk-driven nsclc: first-generation or next-generation tki? Nat Rev Clin Oncol (2018) 15(11):694–708. doi: 10.1038/s41571-018-0081-4 30108370

[2] Yang Z Hackshaw A Feng Q Fu X Zhang Y Mao C. Comparison of gefitinib, erlotinib and afatinib in non-small cell lung cancer: A meta-analysis. Int J Cancer (2017) 140(12):2805–19. doi: 10.1002/ijc.30691 28295308

[3] Soria JC Ohe Y Vansteenkiste J Reungwetwattana T Chewaskulyong B Lee KH. Osimertinib in untreated egfr-mutated advanced non-small-cell lung cancer. N Engl J Med (2018) 378(2):113–25. doi: 10.1056/NEJMoa1713137 29151359

[4] Huang L Fu L. Mechanisms of resistance to egfr tyrosine kinase inhibitors. Acta Pharm Sin B (2015) 5(5):390–401. doi: 10.1016/j.apsb.2015.07.001 26579470 PMC4629442

[5] Shaikh M Shinde Y Pawara R Noolvi M Surana S Ahmad I. Emerging approaches to overcome acquired drug resistance obstacles to osimertinib in non-small-cell lung cancer. J Med Chem (2022) 65(2):1008–46. doi: 10.1021/acs.jmedchem.1c00876 34323489

[6] Zhang B Zhang Y Zhao J Wang Z Wu T Ou W. M2-polarized macrophages contribute to the decreased sensitivity of egfr-tkis treatment in patients with advanced lung adenocarcinoma. Med Oncol (2014) 31(8):127. doi: 10.1007/s12032-014-0127-0 25034365

[7] Wang DH Lee HS Yoon D Berry G Wheeler TM Sugarbaker DJ. Progression of egfr-mutant lung adenocarcinoma is driven by alveolar macrophages. Clin Cancer Res (2017) 23(3):778–88. doi: 10.1158/1078-0432.CCR-15-2597 27496865

[8] Chung FT Lee KY Wang CW Heh CC Chan YF Chen HW. Tumor-associated macrophages correlate with response to epidermal growth factor receptor-tyrosine kinase inhibitors in advanced non-small cell lung cancer. Int J Cancer (2012) 131(3):E227–35. doi: 10.1002/ijc.27403 22174092

[9] Zhang J Li H Wu Q Chen Y Deng Y Yang Z. Tumoral nox4 recruits M2 tumor-associated macrophages *via* ros/pi3k signaling-dependent various cytokine production to promote nsclc growth. Redox Biol (2019) 22:101116. doi: 10.1016/j.redox.2019.101116 30769285 PMC6374999

[10] Casanova-Acebes M Dalla E Leader AM LeBerichel J Nikolic J Morales BM. Tissue-resident macrophages provide a pro-tumorigenic niche to early nsclc cells. Nature (2021) 595(7868):578–84. doi: 10.1038/s41586-021-03651-8 PMC8923521 34135508

[11] Perdiguero EG Geissmann F. The development and maintenance of resident macrophages. Nat Immunol (2016) 17(1):2–8. doi: 10.1038/ni.3341 26681456 PMC4950995

[12] Lavin Y Mortha A Rahman A Merad M. Regulation of macrophage development and function in peripheral tissues. Nat Rev Immunol (2015) 15(12):731–44. doi: 10.1038/nri3920 PMC4706379 26603899

[13] Loyher PL Hamon P Laviron M Meghraoui-Kheddar A Goncalves E Deng Z. Macrophages of distinct origins contribute to tumor development in the lung. J Exp Med (2018) 215(10):2536–53. doi: 10.1084/jem.20180534 PMC6170177 30201786

[14] Murray PJ. Macrophage polarization. Annu Rev Physiol (2017) 79:541–66. doi: 10.1146/annurev-physiol-022516-034339 27813830

[15] Anderson NR Minutolo NG Gill S Klichinsky M. Macrophage-based approaches for cancer immunotherapy. Cancer Res (2021) 81(5):1201–8. doi: 10.1158/0008-5472.Can-20-2990 33203697

[16] Shapouri-Moghaddam A Mohammadian S Vazini H Taghadosi M Esmaeili SA Mardani F. Macrophage plasticity, polarization, and function in health and disease. J Cell Physiol (2018) 233(9):6425–40. doi: 10.1002/jcp.26429 PMC13482560 29319160

[17] Yuan S Chen W Yang J Zheng Y Ye W Xie H. Tumor-associated macrophage-derived exosomes promote egfr-tki resistance in non-small cell lung cancer by regulating the akt, erk1/2 and stat3 signaling pathways. Oncol Lett (2022) 24(4):356. doi: 10.3892/ol.2022.13476 36168315 PMC9478622

[18] Li H Luo F Jiang X Zhang W Xiang T Pan Q. Circitgb6 promotes ovarian cancer cisplatin resistance by resetting tumor-associated macrophage polarization toward the M2 phenotype. J Immunother Cancer (2022) 10(3):e004029–e43. doi: 10.1136/jitc-2021-004029 PMC8919471 35277458

[19] Zhou L Li J Liao M Zhang Q Yang M. Lncrna mir155hg induces M2 macrophage polarization and drug resistance of colorectal cancer cells by regulating anxa2. Cancer Immunol Immunother (2022) 71(5):1075–91. doi: 10.1007/s00262-021-03055-7 PMC10991596 34562123

[20] Niu X Ma J Li J Gu Y Yin L Wang Y. Sodium/glucose cotransporter 1-dependent metabolic alterations induce tamoxifen resistance in breast cancer by promoting macrophage M2 polarization. Cell Death Dis (2021) 12(6):509. doi: 10.1038/s41419-021-03781-x 34006822 PMC8131586

[21] Huang X He C Lin G Lu L Xing K Hua X. Induced cd10 expression during monocyte-to-macrophage differentiation identifies a unique subset of macrophages in pancreatic ductal adenocarcinoma. Biochem Biophys Res Commun (2020) 524(4):1064–71. doi: 10.1016/j.bbrc.2020.02.042 32070494

[22] Wu H Zhang X Han D Cao J Tian J. Tumour-associated macrophages mediate the invasion and metastasis of bladder cancer cells through cxcl8. PeerJ (2020) 8:e8721. doi: 10.7717/peerj.8721 32201645 PMC7073239

[23] Zhang X Chen L Dang WQ Cao MF Xiao JF Lv SQ. Ccl8 secreted by tumor-associated macrophages promotes invasion and stemness of glioblastoma cells *via* erk1/2 signaling. Lab Invest (2020) 100(4):619–29. doi: 10.1038/s41374-019-0345-3 31748682

[24] El-Arabey AA Denizli M Kanlikilicer P Bayraktar R Ivan C Rashed M. Gata3 as a master regulator for interactions of tumor-associated macrophages with high-grade serous ovarian carcinoma. Cell Signal (2020) 68:109539. doi: 10.1016/j.cellsig.2020.109539 31935430

[25] Zhang QW Liu L Gong CY Shi HS Zeng YH Wang XZ. Prognostic significance of tumor-associated macrophages in solid tumor: A meta-analysis of the literature. PloS One (2012) 7(12):e50946. doi: 10.1371/journal.pone.0050946 23284651 PMC3532403

[26] Feng PH Yu CT Wu CY Lee MJ Lee WH Wang LS. Tumor-associated macrophages in stage iiia pn2 non-small cell lung cancer after neoadjuvant chemotherapy and surgery. Am J Transl Res (2014) 6(5):593–603. 25360223 PMC4212933

[27] Dai F Liu L Che G Yu N Pu Q Zhang S. The number and microlocalization of tumor-associated immune cells are associated with patient's survival time in non-small cell lung cancer. BMC Cancer (2010) 10:220. doi: 10.1186/1471-2407-10-220 20487543 PMC2880994

[28] Wu K Lin K Li X Yuan X Xu P Ni P. Redefining tumor-associated macrophage subpopulations and functions in the tumor microenvironment. Front Immunol (2020) 11:1731. doi: 10.3389/fimmu.2020.01731 32849616 PMC7417513

[29] Jia Y Li X Jiang T Zhao S Zhao C Zhang L. Egfr-targeted therapy alters the tumor microenvironment in egfr-driven lung tumors: implications for combination therapies. Int J Cancer (2019) 145(5):1432–44. doi: 10.1002/ijc.32191 30784054

[30] Wu AA Drake V Huang HS Chiu S Zheng L. Reprogramming the tumor microenvironment: tumor-induced immunosuppressive factors paralyze T cells. Oncoimmunology (2015) 4(7):e1016700. doi: 10.1080/2162402x.2015.1016700 26140242 PMC4485788

[31] Parker KH Beury DW Ostrand-Rosenberg S. Myeloid-derived suppressor cells: critical cells driving immune suppression in the tumor microenvironment. Adv Cancer Res (2015) 128:95–139. doi: 10.1016/bs.acr.2015.04.002 26216631 PMC4662416

[32] Liu Q Yu S Zhao W Qin S Chu Q Wu K. Egfr-tkis resistance *via* egfr-independent signaling pathways. Mol Cancer (2018) 17(1):53–61. doi: 10.1186/s12943-018-0793-1 29455669 PMC5817859

[33] Yuan S Dong Y Peng L Yang M Niu L Liu Z. Tumor-associated macrophages affect the biological behavior of lung adenocarcinoma A549 cells through the pi3k/akt signaling pathway. Oncol Lett (2019) 18(2):1840–6. doi: 10.3892/ol.2019.10483 PMC6607053 31423252

[34] Xiao F Liu N Ma X Qin J Liu Y Wang X. M2 macrophages reduce the effect of gefitinib by activating akt/mtor in gefitinib-resistant cell lines hcc827/gr. Thorac Cancer (2020) 11(11):3289–98. doi: 10.1111/1759-7714.13670 PMC7606002 32956565

[35] Wan X Xie B Sun H Gu W Wang C Deng Q. Exosomes derived from M2 type tumor-associated macrophages promote osimertinib resistance in non-small cell lung cancer through mstrg.292666.16-mir-6836-5p-mapk8ip3 axis. Cancer Cell Int (2022) 22(1):83. doi: 10.1186/s12935-022-02509-x 35168607 PMC8845243

[36] Feng PH Yu CT Chen KY Luo CS Wu SM Liu CY. S100a9(+) mdsc and tam-mediated egfr-tki resistance in lung adenocarcinoma: the role of relb. Oncotarget (2018) 9(7):7631–43. doi: 10.18632/oncotarget.24146 PMC5800931 29484139

[37] Gou Y Li X Li P Zhang H Xu T Wang H. Estrogen receptor beta upregulates ccl2 *via* nf-kappab signaling in endometriotic stromal cells and recruits macrophages to promote the pathogenesis of endometriosis. Hum Reprod (2019) 34(4):646–58. doi: 10.1093/humrep/dez019 30838396

[38] Mu J Sun P Ma Z Sun P. Brd4 promotes tumor progression and nf-kappab/ccl2-dependent tumor-associated macrophage recruitment in gist. Cell Death Dis (2019) 10(12):935. doi: 10.1038/s41419-019-2170-4 31819043 PMC6901583

[39] Han R Gu S Zhang Y Luo A Jing X Zhao L. Estrogen promotes progression of hormone-dependent breast cancer through ccl2-ccr2 axis by upregulation of twist *via* pi3k/akt/nf-kappab signaling. Sci Rep (2018) 8(1):9575. doi: 10.1038/s41598-018-27810-6 29934505 PMC6015029

[40] Hua H Kong Q Zhang H Wang J Luo T Jiang Y. Targeting mtor for cancer therapy. J Hematol Oncol (2019) 12(1):71–89. doi: 10.1186/s13045-019-0754-1 31277692 PMC6612215

[41] Saxton RA Sabatini DM. Mtor signaling in growth, metabolism, and disease. Cell (2017) 168(6):960–76. doi: 10.1016/j.cell.2017.02.004 PMC5394987 28283069

[42] Murugan AK. Mtor: role in cancer, metastasis and drug resistance. Semin Cancer Biol (2019) 59:92–111. doi: 10.1016/j.semcancer.2019.07.003 31408724

[43] Yu J Zhang L Peng J Ward R Hao P Wang J. Dictamnine, a novel C-met inhibitor, suppresses the proliferation of lung cancer cells by downregulating the pi3k/akt/mtor and mapk signaling pathways. Biochem Pharmacol (2022) 195:114864. doi: 10.1016/j.bcp.2021.114864 34861243

[44] Hu Y Zhang J Liu Q Ke M Li J Suo W. Torin2 inhibits the egfr-tki resistant non-small lung cancer cell proliferation through negative feedback regulation of akt/mtor signaling. J Cancer (2020) 11(19):5746–57. doi: 10.7150/jca.37417 PMC7477446 32913468

[45] Ishikawa D Takeuchi S Nakagawa T Sano T Nakade J Nanjo S. Mtor inhibitors control the growth of egfr mutant lung cancer even after acquiring resistance by hgf. PloS One (2013) 8(5):e62104. doi: 10.1371/journal.pone.0062104 23690929 PMC3653905

[46] Wu YY Wu HC Wu JE Huang KY Yang SC Chen SX. The dual pi3k/mtor inhibitor bez235 restricts the growth of lung cancer tumors regardless of egfr status, as a potent accompanist in combined therapeutic regimens. J Exp Clin Cancer Res (2019) 38(1):282. doi: 10.1186/s13046-019-1282-0 31262325 PMC6604380

[47] Lai IC Lai GM Chow JM Lee HL Yeh CF Li CH. Active fraction (Hs7) from Taiwanofungus camphoratus inhibits akt-mtor, erk and stat3 pathways and induces cdk inhibitors in cl1-0 human lung cancer cells. Chin Med (2017) 12:33. doi: 10.1186/s13020-017-0154-9 29177004 PMC5688709

[48] Dong S Zhang XC Cheng H Zhu JQ Chen ZH Zhang YF. Everolimus synergizes with gefitinib in non-small-cell lung cancer cell lines resistant to epidermal growth factor receptor tyrosine kinase inhibitors. Cancer Chemother Pharmacol (2012) 70(5):707–16. doi: 10.1007/s00280-012-1946-3 22941374

[49] Sano T Takeuchi S Nakagawa T Ishikawa D Nanjo S Yamada T. The novel phosphoinositide 3-kinase-mamMalian target of rapamycin inhibitor, bez235, circumvents erlotinib resistance of epidermal growth factor receptor mutant lung cancer cells triggered by hepatocyte growth factor. Int J Cancer (2013) 133(2):505–13. doi: 10.1002/ijc.28034 23319394

[50] Fei SJ Zhang XC Dong S Cheng H Zhang YF Huang L. Targeting mtor to overcome epidermal growth factor receptor tyrosine kinase inhibitor resistance in non-small cell lung cancer cells. PloS One (2013) 8(7):e69104. doi: 10.1371/journal.pone.0069104 23874880 PMC3712950

[51] Wang G Zhao J Zhang M Wang Q Chen B Hou Y. Ferumoxytol and cpg oligodeoxynucleotide 2395 synergistically enhance antitumor activity of macrophages against nsclc with egfr(L858r/T790m) mutation. Int J Nanomed (2019) 14:4503–15. doi: 10.2147/IJN.S193583 PMC6599896 31417255

[52] Qu Y Wu X Yin Y Yang Y Ma D Li H. Antitumor activity of selective mek1/2 inhibitor azd6244 in combination with pi3k/mtor inhibitor bez235 in gefitinib-resistant nsclc xenograft models. J Exp Clin Cancer Res (2014) 33(1):52. doi: 10.1186/1756-9966-33-52 24939055 PMC4074836

[53] Moran T Palmero R Provencio M Insa A Majem M Reguart N. A phase ib trial of continuous once-daily oral afatinib plus sirolimus in patients with epidermal growth factor receptor mutation-positive non-small cell lung cancer and/or disease progression following prior erlotinib or gefitinib. Lung Cancer (2017) 108:154–60. doi: 10.1016/j.lungcan.2017.03.009 28625629

[54] Yip PY. Phosphatidylinositol 3-kinase-akt-mamMalian target of rapamycin (Pi3k-akt-mtor) signaling pathway in non-small cell lung cancer. Transl Lung Cancer Res (2015) 4(2):165–76. doi: 10.3978/j.issn.2218-6751.2015.01.04 PMC4384220 25870799

[55] Botting GM Rastogi I Chhabra G Nlend M Puri N. Mechanism of resistance and novel targets mediating resistance to egfr and C-met tyrosine kinase inhibitors in non-small cell lung cancer. PloS One (2015) 10(8):e0136155. doi: 10.1371/journal.pone.0136155 26301867 PMC4547756

[56] He C Zheng S Luo Y Wang B. Exosome theranostics: biology and translational medicine. Theranostics (2018) 8(1):237–55. doi: 10.7150/thno.21945 PMC5743472 29290805

[57] Kalluri R LeBleu VS. The biology, function, and biomedical applications of exosomes. Science (2020) 367(6478):6977–91. doi: 10.1126/science.aau6977 PMC7717626 32029601

[58] Bauer AK Velmurugan K Xiong KN Alexander CM Xiong J Brooks R. Epiregulin is required for lung tumor promotion in a murine two-stage carcinogenesis model. Mol Carcinog (2017) 56(1):94–105. doi: 10.1002/mc.22475 26894620 PMC5575741

[59] Ma S Zhang L Ren Y Dai W Chen T Luo L. Epiregulin confers egfr-tki resistance *via* egfr/erbb2 heterodimer in non-small cell lung cancer. Oncogene (2021) 40(14):2596–609. doi: 10.1038/s41388-021-01734-4 33750895

[60] Wang X Xu J Chen J Jin S Yao J Yu T. Il-22 confers egfr-tki resistance in nsclc *via* the akt and erk signaling pathways. Front Oncol (2019) 9:1167. doi: 10.3389/fonc.2019.01167 31750252 PMC6848259

[61] Lu GS Li M Xu CX Wang D. Ape1 stimulates egfr-tki resistance by activating akt signaling through a redox-dependent mechanism in lung adenocarcinoma. Cell Death Dis (2018) 9(11):1111. doi: 10.1038/s41419-018-1162-0 30382076 PMC6208429

[62] Wu Y Yu DD Hu Y Cao HX Yu SR Liu SW. Lxr ligands sensitize egfr-tki-resistant human lung cancer cells *in vitro* by inhibiting akt activation. Biochem Biophys Res Commun (2015) 467(4):900–5. doi: 10.1016/j.bbrc.2015.10.047 26471306

[63] Kang XH Xu ZY Gong YB Wang LF Wang ZQ Xu L. Bufalin reverses hgf-induced resistance to egfr-tkis in egfr mutant lung cancer cells *via* blockage of met/pi3k/akt pathway and induction of apoptosis. Evid Based Complement Alternat Med (2013) 2013:243859. doi: 10.1155/2013/243859 23533466 PMC3603503

[64] Bokobza SM Jiang Y Weber AM Devery AM Ryan AJ. Combining akt inhibition with chloroquine and gefitinib prevents compensatory autophagy and induces cell death in egfr mutated nsclc cells. Oncotarget (2014) 5(13):4765–78. doi: 10.18632/oncotarget.2017 PMC4148097 24946858

[65] Wu H Fan F Liu Z Shen C Wang A Lu Y. Norcantharidin combined with egfr-tkis overcomes hgf-induced resistance to egfr-tkis in egfr mutant lung cancer cells *via* inhibition of met/pi3k/akt pathway. Cancer Chemother Pharmacol (2015) 76(2):307–15. doi: 10.1007/s00280-015-2792-x 26063323

[66] Meng F Wang F Wang L Wong SC Cho WC Chan LW. Mir-30a-5p overexpression may overcome egfr-inhibitor resistance through regulating pi3k/akt signaling pathway in non-small cell lung cancer cell lines. Front Genet (2016) 7:197. doi: 10.3389/fgene.2016.00197 27895663 PMC5108768

[67] Xie M He J He C Wei S. Gamma secretase inhibitor bms-708163 reverses resistance to egfr inhibitor *via* the pi3k/akt pathway in lung cancer. J Cell Biochem (2015) 116(6):1019–27. doi: 10.1002/jcb.25056 25561332

[68] Lai L Shen Q Wang Y Chen L Lai J Wu Z. Polyphyllin I reverses the resistance of osimertinib in non-small cell lung cancer cell through regulation of pi3k/akt signaling. Toxicol Appl Pharmacol (2021) 419:115518. doi: 10.1016/j.taap.2021.115518 33812963

[69] Wang F Meng F Wong SCC Cho WCS Yang S Chan LWC. Combination therapy of gefitinib and mir-30a-5p may overcome acquired drug resistance through regulating the pi3k/akt pathway in non-small cell lung cancer. Ther Adv Respir Dis (2020) 14:1753466620915156. doi: 10.1177/1753466620915156 32552611 PMC7303773

[70] Clément-Duchêne C Natale RB Jahan T Krupitskaya Y Osarogiagbon R Sanborn RE. A phase ii study of enzastaurin in combination with erlotinib in patients with previously treated advanced non-small cell lung cancer. Lung Cancer (2012) 78(1):57–62. doi: 10.1016/j.lungcan.2012.06.003 22809813

[71] Noto A De Vitis C Roscilli G Fattore L Malpicci D Marra E. Combination therapy with anti-erbb3 monoclonal antibodies and egfr tkis potently inhibits non-small cell lung cancer. Oncotarget (2013) 4(8):1253–65. doi: 10.18632/oncotarget.1141 PMC3787155 23896512

[72] Si Y Pei X Wang X Han Q Xu C Zhang B. An anti-egfr/anti- her2 bispecific antibody with enhanced antitumor activity against acquired gefitinib-resistant nsclc cells. Protein Pept Lett (2021) 28(11):1290–7. doi: 10.2174/0929866528666210930170624 34602035

[73] Peng H Chen B Huang W Tang Y Jiang Y Zhang W. Reprogramming tumor-associated macrophages to reverse egfr(T790m) resistance by dual-targeting codelivery of gefitinib/vorinostat. Nano Lett (2017) 17(12):7684–90. doi: 10.1021/acs.nanolett.7b03756 29160717

[74] Gori S Foglietta J Mameli MG Stocchi L Fenocchio D Anastasi P. Her-3 status by immunohistochemistry in her-2-positive metastatic breast cancer patients treated with trastuzumab: correlation with clinical outcome. Tumori (2012) 98(1):39–44. doi: 10.1177/030089161209800105 22495700

[75] Vicencio JM Evans R Green R An Z Deng J Treacy C. Osimertinib and anti-her3 combination therapy engages immune dependent tumor toxicity *via* sting activation in trans. Cell Death Dis (2022) 13(3):274. doi: 10.1038/s41419-022-04701-3 35347108 PMC8960767

[76] Yan H Bu P. Non-coding rna in cancer. Essays Biochem (2021) 65(4):625–39. doi: 10.1042/ebc20200032 PMC8564738 33860799

[77] Deng Q Fang Q Xie B Sun H Bao Y Zhou S. Exosomal long non-coding rna mstrg.292666.16 is associated with osimertinib (Azd9291) resistance in non-small cell lung cancer. Aging (Albany NY) (2020) 12(9):8001–15. doi: 10.18632/aging.103119 PMC7244069 32375124

[78] Gabrilovich DI. Myeloid-derived suppressor cells. Cancer Immunol Res (2017) 5(1):3–8. doi: 10.1158/2326-6066.Cir-16-0297 28052991 PMC5426480

[79] Hegde S Leader AM Merad M. Mdsc: markers, development, states, and unaddressed complexity. Immunity (2021) 54(5):875–84. doi: 10.1016/j.immuni.2021.04.004 PMC8709560 33979585

[80] Mirzaei S Saghari S Bassiri F Raesi R Zarrabi A Hushmandi K. Nf-kappab as a regulator of cancer metastasis and therapy response: A focus on epithelial-mesenchymal transition. J Cell Physiol (2022) 237(7):2770–95. doi: 10.1002/jcp.30759 35561232

[81] Yeo MK Kim Y Lee DH Chung C Bae GE. Cosuppression of nf-kappab and aicda overcomes acquired egfr-tki resistance in non-small cell lung cancer. Cancers (Basel) (2022) 14(12):2940–50. doi: 10.3390/cancers14122940 PMC9221089 35740609

[82] Liu S Cao H Chen D Yu S Sha H Wu J. Lxr ligands induce apoptosis of egfr-tki-resistant human lung cancer cells *in vitro* by inhibiting akt-nf-kappab activation. Oncol Lett (2018) 15(5):7168–74. doi: 10.3892/ol.2018.8182 PMC5921072 29731879

[83] Zhang Y Zhu S Du Y Xu F Sun W Xu Z. Relb upregulates pd-L1 and exacerbates prostate cancer immune evasion. J Exp Clin Cancer Res (2022) 41(1):66–81. doi: 10.1186/s13046-022-02243-2 35177112 PMC8851785

[84] Peng S Wang R Zhang X Ma Y Zhong L Li K. Egfr-tki resistance promotes immune escape in lung cancer *via* increased pd-L1 expression. Mol Cancer (2019) 18(1):165. doi: 10.1186/s12943-019-1073-4 31747941 PMC6864970

[85] Sugiyama E Togashi Y Takeuchi Y Shinya S Tada Y Kataoka K. Blockade of egfr improves responsiveness to pd-1 blockade in egfr-mutated non-small cell lung cancer. Sci Immunol (2020) 5(43):3937–49. doi: 10.1126/sciimmunol.aav3937 32005679

[86] Gurule NJ McCoach CE Hinz TK Merrick DT Van Bokhoven A Kim J. A tyrosine kinase inhibitor-induced interferon response positively associates with clinical response in egfr-mutant lung cancer. NPJ Precis Oncol (2021) 5(1):41. doi: 10.1038/s41698-021-00181-4 34001994 PMC8129124

[87] Ruffell B Chang-Strachan D Chan V Rosenbusch A Ho CM Pryer N. Macrophage il-10 blocks cd8+ T cell-dependent responses to chemotherapy by suppressing il-12 expression in intratumoral dendritic cells. Cancer Cell (2014) 26(5):623–37. doi: 10.1016/j.ccell.2014.09.006 PMC4254570 25446896

[88] Salvagno C Ciampricotti M Tuit S Hau CS van Weverwijk A Coffelt SB. Therapeutic targeting of macrophages enhances chemotherapy efficacy by unleashing type I interferon response. Nat Cell Biol (2019) 21(4):511–21. doi: 10.1038/s41556-019-0298-1 PMC6451630 30886344

[89] Ruffell B Coussens LM. Macrophages and therapeutic resistance in cancer. Cancer Cell (2015) 27(4):462–72. doi: 10.1016/j.ccell.2015.02.015 PMC4400235 25858805

[90] Nixon BG Kuo F Ji L Liu M Capistrano K Do M. Tumor-associated macrophages expressing the transcription factor irf8 promote T cell exhaustion in cancer. Immunity (2022) 55(11):2044–58 e5. doi: 10.1016/j.immuni.2022.10.002 36288724 PMC9649891

[91] Lin Z Wang Q Jiang T Wang W Zhao JJ. Targeting tumor-associated macrophages with sting agonism improves the antitumor efficacy of osimertinib in a mouse model of egfr-mutant lung cancer. Front Immunol (2023) 14:1077203. doi: 10.3389/fimmu.2023.1077203 36817465 PMC9933873

[92] Zhang Z Zhou H Ouyang X Dong Y Sarapultsev A Luo S. Multifaceted functions of sting in human health and disease: from molecular mechanism to targeted strategy. Signal Transduct Target Ther (2022) 7(1):394–422. doi: 10.1038/s41392-022-01252-z 36550103 PMC9780328

[93] Perrotta C Cervia D Di Renzo I Moscheni C Bassi MT Campana L. Nitric oxide generated by tumor-associated macrophages is responsible for cancer resistance to cisplatin and correlated with syntaxin 4 and acid sphingomyelinase inhibition. Front Immunol (2018) 9:1186. doi: 10.3389/fimmu.2018.01186 29896202 PMC5987706

[94] Navasardyan I Bonavida B. Regulation of T cells in cancer by nitric oxide. Cells (2021) 10(10):2655–76. doi: 10.3390/cells10102655 PMC8534057 34685635

[95] Shinchi Y Ishizuka S Komohara Y Matsubara E Mito R Pan C. The expression of pd-1 ligand 1 on macrophages and its clinical impacts and mechanisms in lung adenocarcinoma. Cancer Immunol Immunother (2022) 71(11):2645–61. doi: 10.1007/s00262-022-03187-4 PMC8963674 35352168

[96] Sumitomo R Hirai T Fujita M Murakami H Otake Y Huang CL. Pd-L1 expression on tumor-infiltrating immune cells is highly associated with M2 tam and aggressive MALIgnant potential in patients with resected non-small cell lung cancer. Lung Cancer (2019) 136:136–44. doi: 10.1016/j.lungcan.2019.08.023 31499335

[97] Ding L Kim HJ Wang Q Kearns M Jiang T Ohlson CE. Parp inhibition elicits sting-dependent antitumor immunity in brca1-deficient ovarian cancer. Cell Rep (2018) 25(11):2972–80 e5. doi: 10.1016/j.celrep.2018.11.054 30540933 PMC6366450

[98] Yang H Lee WS Kong SJ Kim CG Kim JH Chang SK. Sting activation reprograms tumor vasculatures and synergizes with vegfr2 blockade. J Clin Invest (2019) 129(10):4350–64. doi: 10.1172/JCI125413 PMC6763266 31343989

[99] Xiong H Mittman S Rodriguez R Moskalenko M Pacheco-Sanchez P Yang Y. Anti-pd-L1 treatment results in functional remodeling of the macrophage compartment. Cancer Res (2019) 79(7):1493–506. doi: 10.1158/0008-5472.Can-18-3208 30679180

[100] Ahn MJ Cho BC Ou X Walding A Dymond AW Ren S. Osimertinib plus durvalumab in patients with egfr-mutated, advanced nsclc: A phase 1b, open-label, multicenter trial. J Thorac Oncol (2022) 17(5):718–23. doi: 10.1016/j.jtho.2022.01.012 35181499

[101] Batista-Gonzalez A Vidal R Criollo A Carreño LJ. New insights on the role of lipid metabolism in the metabolic reprogramming of macrophages. Front Immunol (2019) 10:2993. doi: 10.3389/fimmu.2019.02993 31998297 PMC6966486

[102] Chen Z Yu D Owonikoko TK RaMalingam SS Sun SY. Induction of srebp1 degradation coupled with suppression of srebp1-mediated lipogenesis impacts the response of egfr mutant nsclc cells to osimertinib. Oncogene (2021) 40(49):6653–65. doi: 10.1038/s41388-021-02057-0 PMC8671366 34635799

[103] Liang L He H Jiang S Liu Y Huang J Sun X. Tiam2 contributes to osimertinib resistance, cell motility, and tumor-associated macrophage M2-like polarization in lung adenocarcinoma. Int J Mol Sci (2022) 23(18):10415–34. doi: 10.3390/ijms231810415 PMC9499457 36142328

[104] Jin H He Y Zhao P Hu Y Tao J Chen J. Targeting lipid metabolism to overcome emt-associated drug resistance *via* integrin beta3/fak pathway and tumor-associated macrophage repolarization using legumain-activatable delivery. Theranostics (2019) 9(1):265–78. doi: 10.7150/thno.27246 PMC6332796 30662566

[105] Yin W Zhao Y Kang X Zhao P Fu X Mo X. Bbb-penetrating codelivery liposomes treat brain metastasis of non-small cell lung cancer with egfr(T790m) mutation. Theranostics (2020) 10(14):6122–35. doi: 10.7150/thno.42234 PMC7255027 32483443

[106] Chen K Zhou F Shen W Jiang T Wu X Tong X. Novel mutations on egfr leu792 potentially correlate to acquired resistance to osimertinib in advanced nsclc. J Thorac Oncol (2017) 12(6):e65–e8. doi: 10.1016/j.jtho.2016.12.024 28093244

[107] Sun Y Dong Y Liu X Zhang Y Bai H Duan J. Blockade of stat3/il-4 overcomes egfr T790m-cis-L792f-induced resistance to osimertinib *via* suppressing M2 macrophages polarization. EBioMedicine (2022) 83:104200. doi: 10.1016/j.ebiom.2022.104200 35932642 PMC9358434

[108] Wu K Chang Q Lu Y Qiu P Chen B Thakur C. Gefitinib resistance resulted from stat3-mediated akt activation in lung cancer cells. Oncotarget (2013) 4(12):2430–8. doi: 10.18632/oncotarget.1431 PMC3926838 24280348

[109] Park HJ Park SH Choi YH Chi GY. The root extract of scutellaria baicalensis induces apoptosis in egfr tki-resistant human lung cancer cells by inactivation of stat3. Int J Mol Sci (2021) 22(10):5181–97. doi: 10.3390/ijms22105181 34068421 PMC8153615

[110] Shu D Xu Y Chen W. Knockdown of lncrna blacat1 reverses the resistance of afatinib to non-small cell lung cancer *via* modulating stat3 signalling. J Drug Target (2020) 28(3):300–6. doi: 10.1080/1061186x.2019.1650368 31359792

[111] Lu J Li J Lin Z Li H Lou L Ding W. Reprogramming of tams *via* the stat3/cd47-sirpalpha axis promotes acquired resistance to egfr-tkis in lung cancer. Cancer Lett (2023) 564:216205. doi: 10.1016/j.canlet.2023.216205 37146936

[112] Dong J Cheng XD Zhang WD Qin JJ. Recent update on development of small-molecule stat3 inhibitors for cancer therapy: from phosphorylation inhibition to protein degradation. J Med Chem (2021) 64(13):8884–915. doi: 10.1021/acs.jmedchem.1c00629 34170703

[113] Zou S Tong Q Liu B Huang W Tian Y Fu X. Targeting stat3 in cancer immunotherapy. Mol Cancer (2020) 19(1):145. doi: 10.1186/s12943-020-01258-7 32972405 PMC7513516

[114] Zhou D Xia Z Xie M Gao Y Yu Q He B. Exosomal long non-coding rna sox2 overlapping transcript enhances the resistance to egfr-tkis in non-small cell lung cancer cell line H1975. Hum Cell (2021) 34(5):1478–89. doi: 10.1007/s13577-021-00572-6 34244990

[115] Zhao B Zhang Y Lu S Li M. Macrophage renewal modes affect acquired resistance to gefitinib in egfr−Mutant lung cancer pc−9 cells. Oncol Rep (2023) 49(2):30–40. doi: 10.3892/or.2022.8467 36562379

[116] Low-Marchelli JM Ardi VC Vizcarra EA van Rooijen N Quigley JP Yang J. Twist1 induces ccl2 and recruits macrophages to promote angiogenesis. Cancer Res (2013) 73(2):662–71. doi: 10.1158/0008-5472.Can-12-0653 PMC3566985 23329645

[117] Mantovani A Marchesi F Malesci A Laghi L Allavena P. Tumour-associated macrophages as treatment targets in oncology. Nat Rev Clin Oncol (2017) 14(7):399–416. doi: 10.1038/nrclinonc.2016.217 28117416 PMC5480600

[118] Cannarile MA Weisser M Jacob W Jegg AM Ries CH Rüttinger D. Colony-stimulating factor 1 receptor (Csf1r) inhibitors in cancer therapy. J Immunother Cancer (2017) 5(1):53. doi: 10.1186/s40425-017-0257-y 28716061 PMC5514481

[119] Sidorov M Dighe P Woo RWL Rodriguez-Brotons A Chen M Ice RJ. Dual targeting of egfr and mtor pathways inhibits glioblastoma growth by modulating the tumor microenvironment. Cells (2023) 12(4):547–65. doi: 10.3390/cells12040547 PMC9954001 36831214

[120] Schmall A Al-Tamari HM Herold S Kampschulte M Weigert A Wietelmann A. Macrophage and cancer cell cross-talk *via* ccr2 and cx3cr1 is a fundamental mechanism driving lung cancer. Am J Respir Crit Care Med (2015) 191(4):437–47. doi: 10.1164/rccm.201406-1137OC 25536148

[121] Sawa-Wejksza K Kandefer-Szerszeń M. Tumor-associated macrophages as target for antitumor therapy. Arch Immunol Ther Exp (Warsz) (2018) 66(2):97–111. doi: 10.1007/s00005-017-0480-8 28660349 PMC5851686

[122] Lamouille S Xu J Derynck R. Molecular mechanisms of epithelial-mesenchymal transition. Nat Rev Mol Cell Biol (2014) 15(3):178–96. doi: 10.1038/nrm3758 PMC4240281 24556840

[123] Buonato JM Lazzara MJ. Erk1/2 blockade prevents epithelial-mesenchymal transition in lung cancer cells and promotes their sensitivity to egfr inhibition. Cancer Res (2014) 74(1):309–19. doi: 10.1158/0008-5472.Can-12-4721 PMC3964587 24108744

[124] Tulchinsky E Demidov O Kriajevska M Barlev NA Imyanitov E. Emt: A mechanism for escape from egfr-targeted therapy in lung cancer. Biochim Biophys Acta Rev Cancer (2019) 1871(1):29–39. doi: 10.1016/j.bbcan.2018.10.003 30419315

[125] Nilsson MB Yang Y Heeke S Patel SA Poteete A Udagawa H. Cd70 is a therapeutic target upregulated in emt-associated egfr tyrosine kinase inhibitor resistance. Cancer Cell (2023) 41(2):340–55.e6. doi: 10.1016/j.ccell.2023.01.007 36787696 PMC10259078

[126] Raoof S Mulford IJ Frisco-Cabanos H Nangia V Timonina D Labrot E. Targeting fgfr overcomes emt-mediated resistance in egfr mutant non-small cell lung cancer. Oncogene (2019) 38(37):6399–413. doi: 10.1038/s41388-019-0887-2 PMC6742540 31324888

[127] Ji W Choi YJ Kang MH Sung KJ Kim DH Jung S. Efficacy of the cdk7 inhibitor on emt-associated resistance to 3rd generation egfr-tkis in non-small cell lung cancer cell lines. Cells (2020) 9(12):2596–609. doi: 10.3390/cells9122596 PMC7761809 33287368

[128] Li L Han R Xiao H Lin C Wang Y Liu H. Metformin sensitizes egfr-tki-resistant human lung cancer cells *in vitro* and *in vivo* through inhibition of il-6 signaling and emt reversal. Clin Cancer Res (2014) 20(10):2714–26. doi: 10.1158/1078-0432.Ccr-13-2613 24644001

[129] Bonde AK Tischler V Kumar S Soltermann A Schwendener RA. Intratumoral macrophages contribute to epithelial-mesenchymal transition in solid tumors. BMC Cancer (2012) 12:35. doi: 10.1186/1471-2407-12-35 22273460 PMC3314544

[130] Shen M Xu Z Xu W Jiang K Zhang F Ding Q. Inhibition of atm reverses emt and decreases metastatic potential of cisplatin-resistant lung cancer cells through jak/stat3/pd-L1 pathway. J Exp Clin Cancer Res (2019) 38(1):149–62. doi: 10.1186/s13046-019-1161-8 PMC6454747 30961670

[131] Karachaliou N Codony-Servat J Teixidó C Pilotto S Drozdowskyj A Codony-Servat C. Bim and mtor expression levels predict outcome to erlotinib in egfr-mutant non-small-cell lung cancer. Sci Rep (2015) 5:17499–510. doi: 10.1038/srep17499 PMC4671004 26639561

[132] Long MP Wang HL Luo YB Yang JH. Targeting ror1 inhibits epithelial to mesenchymal transition in human lung adenocarcinoma *via* mtor signaling pathway. Int J Clin Exp Pathol (2018) 11(10):4759–70. PMC6962910 31949551

[133] Zhang Q Zhang Y Chen Y Qian J Zhang X Yu K. A novel mtorc1/2 inhibitor (Mti-31) inhibits tumor growth, epithelial-mesenchymal transition, metastases, and improves antitumor immunity in preclinical models of lung cancer. Clin Cancer Res (2019) 25(12):3630–42. doi: 10.1158/1078-0432.CCR-18-2548 30796032

[134] Song X Tang W Peng H Qi X Li J. Fgfr leads to sustained activation of stat3 to mediate resistance to egfr-tkis treatment. Invest New Drugs (2021) 39(5):1201–12. doi: 10.1007/s10637-021-01061-1 33829354

[135] Yang Y Wang W Chang H Han Z Yu X Zhang T. Reciprocal regulation of mir-206 and il-6/stat3 pathway mediates il6-induced gefitinib resistance in egfr-mutant lung cancer cells. J Cell Mol Med (2019) 23(11):7331–41. doi: 10.1111/jcmm.14592 PMC6815809 31507089

[136] Kim SM Kwon OJ Hong YK Kim JH Solca F Ha SJ. Activation of il-6r/jak1/stat3 signaling induces *de novo* resistance to irreversible egfr inhibitors in non-small cell lung cancer with T790m resistance mutation. Mol Cancer Ther (2012) 11(10):2254–64. doi: 10.1158/1535-7163.Mct-12-0311 22891040

[137] Zheng Q Dong H Mo J Zhang Y Huang J Ouyang S. A novel stat3 inhibitor W2014-S regresses human non-small cell lung cancer xenografts and sensitizes egfr-tki acquired resistance. Theranostics (2021) 11(2):824–40. doi: 10.7150/thno.49600 PMC7738869 33391507

[138] Wang Q Lu B Zhang Y Yu J Guo J Zhou Q. Stat3 inhibitor bbi608 enhances the antitumor effect of gefitinib on egfr-mutated non-small cell lung cancer cells. Hum Cell (2021) 34(6):1855–65. doi: 10.1007/s13577-021-00582-4 34370268

[139] Parayath NN Parikh A Amiji MM. Repolarization of tumor-associated macrophages in a genetically engineered nonsmall cell lung cancer model by intraperitoneal administration of hyaluronic acid-based nanoparticles encapsulating microrna-125b. Nano Lett (2018) 18(6):3571–9. doi: 10.1021/acs.nanolett.8b00689 29722542

[140] Sarode P Zheng X Giotopoulou GA Weigert A Kuenne C Gunther S. Reprogramming of tumor-associated macrophages by targeting beta-catenin/fosl2/arid5a signaling: A potential treatment of lung cancer. Sci Adv (2020) 6(23):eaaz6105. doi: 10.1126/sciadv.aaz6105 32548260 PMC7274802

